# Supplementary material for: “I’m not gonna be able to do anything about it, then what’s the point?”: A broad group of stakeholders identify barriers and facilitators to HCV testing in a Massachusetts jail
Source: PLoS One. 2021 May 26;16(5):e0250901. doi: 10.1371/journal.pone.0250901 (PMC8153419; doi:10.1371/journal.pone.0250901)
Supplement: S1 Table — This is the codebook used for the interviews conducted with people who were incarcerated. (PDF) [file pone.0250901.s002.pdf]

S1 Table. Codes for People Who Are In Jail.

**Codes**	**Subcodes**
Priorities	
Access	
Barriers	
Legal	Legal action
	Healthcare system
	Legal rights
	HIV vs HCV
Treatment	Treatment
	Medications
	Side effects
	Treatment payment
Status quo	Normalization
	Status quo
	Perceived changes over time
	Institutional inertia
	Inevitability
	Jail is supposed to be bad
	Seriousness
Deservingness	Deservingness
	Risk behaviors
	Mistrust
	Concern for transmission
	Stigma
	Reinfection
	Mandate to treat
	Frustration
Transience	Transient population
Systems perspective	Differential care
	Fragmented healthcare
	Facilitators
	Captive audience
	Linkage to care
Knowledge:	Knowledge
	Word of mouth
	Patient provider communication
	Reliable information
	Conflicting beliefs
	Uncertainty about future

	HCV status
	Personal experience
	Positive personal experience
	Negative personal experience
	Social network
	Personal health
	Does not know people with hcv
	Self-efficacy
	Knows people with hcv
	Control
Testing	Testing
	“”Frequency
	“”payment
	“”Testing
	“”preference
	“”location
