# Supplementary material for: “I’m not gonna be able to do anything about it, then what’s the point?”: A broad group of stakeholders identify barriers and facilitators to HCV testing in a Massachusetts jail
Source: PLoS One. 2021 May 26;16(5):e0250901. doi: 10.1371/journal.pone.0250901 (PMC8153419; doi:10.1371/journal.pone.0250901)
Supplement: S2 Table — This is the codebook used for the interviews conducted with non-incarcerated stakeholders. (PDF) [file pone.0250901.s003.pdf]

S2 Table. Codes for Non-Incarcerated Stakeholders

**Code**	**Sub-Code**
Barriers	
Access	
Testing	
Treatment	
Facilitators	
Cost-related	Limited resources
	Fiscal barriers
	Trade-off between cost and outcome
	Funding
	Priorities (what does the speaker think?)
	Priorities (what does the system value)
	Side-effects
Deservingness	Deservingness
	Obligation
	Mandate to treat
	Jail is supposed to be bad
	Moral responsibility
	Mistrust
	Stigma
	Seriousness
	Frustration
	Risk behaviors
	Substance use disorder treatment
	Reinfection
Transience	Differences between jails/prisons
(issues with transient population	Silo-ed healthcare
from system perspective)	Heterogeneity between jails
	Outsourced/privatized healthcare
	Differential care
	Fragmented healthcare
	HIV vs HCV
	Healthcare systems
	Building on current systems
	Standard of care
	Sentence period
	Complexity of the system
	Transient population
	Captive audience
	Current process
	Uncertainty about future
Public Health	Transmission
	Outbreak
	Harm reduction
	Public health

	HCV prevalence
	Need for data
	Shared responsibility
	Personal responsibility
	Interdisciplinary collaboration
	Linkage to care
	Unclear roles and responsibilities
Legal/political	Historical changes
How does policy influence HCV care?	Change in legal landscape
	Legal action
	Policy
	Suggestions for improvement
	What is working well
	Political process
	Legal rights
Communication	Communication
	Comfort when discussing hcv
	Knowledge
	Word of mouth
	Patient provider communication
	Conflicting beliefs
	Experience
	Educational resources
	Guidelines
Choices	Control
	Normalization
	Inevitability
	Patient autonomy
	Clinician autonomy
	Status quo
	Decision makers
