# Supplementary material for: “I’m not gonna be able to do anything about it, then what’s the point?”: A broad group of stakeholders identify barriers and facilitators to HCV testing in a Massachusetts jail
Source: PLoS One. 2021 May 26;16(5):e0250901. doi: 10.1371/journal.pone.0250901 (PMC8153419; doi:10.1371/journal.pone.0250901)
Supplement: S1 File — These are the questions asked of participants who were incarcerated. (DOCX) [file pone.0250901.s004.docx]

**HCV Care: Qualitative Interview Guide:**

**People Who are In Jail (Interview takes place in Jail)**

Participant ID#: ___ ___ ___

Interview Date: ___ ___ / ___ ___ / ___ ___

M M D D Y Y

Self-Identified Race (eg White, Black, etc):

Self-Identified Ethnicity (Hispanic/Non-Hispanic):

Preferred Language: English Spanish

Age: ___ ___

Self-Identified Gender:

M F Other:___________

***Interviewer:***

*Thank you for agreeing to be a part of this interview. I will ask you a series of questions about Hepatitis C Virus (also known as Hep C).*

*There are no “right” or “wrong” answers to your questions. We want to hear anything that you want to talk about. We are going to be typing notes on the computer when you talk and we may ask you to pause or repeat something. We will not put your name on anything that we type or write.*

*If you do not feel comfortable answering a question, it is ok for you to tell me that you do not want to answer. If there’s a question that you’d prefer not to answer, simply say “pass.” You can also choose to stop this interview at any time and for any reason.*

*Your decision to participate or not will not change your health care or the time you are serving in jail.*

*Before we begin, do you have any questions?*

Time Interview Started: ___ ___ : ___ ___ am / pm

Time Interview Ended: ___ ___ : ___ ___ am / pm

Signature of person performing interview: ___________________________________

**HCV Care: Qualitative Interview Guide:**

**People Who are In Jail (Interview takes place in Jail)**

| **Interviewer Asks Verbatim** | | **Probes (ask these if the participant needs not sure how to answer or is brief in answer)** |
| --- | --- | --- |
| *Thank you for participating in this study. We are interested in getting to know you and learning about your opinions. First, I would like to start by asking, do you have a favorite song or music artist?* | | - Do you remember the first time you heard this song or artist? - Transition: Thank you for sharing that. We look forward to hearing about your opinions. Today we will specifically talk about your thoughts on Hepatitis C. |
| *What have you heard about Hepatitis C?* | | - Does anyone you know (parents, friends, cousins) have Hepatitis C? - When was the first time you heard about Hepatitis C? - How does someone get Hepatitis C? - What does Hep C do to the body? - Can you cure Hep C? |
| *Have you ever been tested for Hep C?* | | - [YES]: - Did you get the results? - Tell me about the experience. Whose idea was it (you, your provider, a friend)? - Did you feel like you had control over getting Hep C testing? - Could you refuse testing? Did you feel like it was pushed on you? - *If you could change the process of Hep C testing, how would you change it?* |
|  |  | - [NO]: - Have you been offered testing but said no? Tell me about your decision |
|  |  | - [NOT SURE]: - Is there a time in your life that you feel you should have been tested for Hep C? |
| *How does someone get tested for Hepatitis C?* | | - What kinds of tests are there (rapid, serum, viral load, genotype)? - Where can you go to get tested (PCP, ED, jail, etc)? |
| *Where does someone get tested for Hepatitis C?* | | - Does it need to happen at a primary care office? Emergency room? In jail or prison? |
| *How often should someone get tested for Hepatitis C?* | | - Where does someone learn about how often Hep C testing should happen? |
| *Who do you think pays for Hep C testing?* | |  |
| *Are there reasons why people should be tested for Hepatitis C?* | |  |
| *Are there reasons why people should not be tested for Hepatitis C?* | |  |
| *Given what you know, how do you feel about Hepatitis C?* | |  |
| *Given what you know, how serious is Hepatitis C as a health condition?* | | - How serious is Hepatitis C in comparison to HIV? TB? Diabetes? Asthma? |
| *Were you ever offered HIV and Hep C testing together?* | | - [YES]: - Did getting offered HIV and Hep C testing together influence your decision to get tested? |
|  |  | - [NO]: - Would getting offered HIV and Hep C testing together influence your decision to get tested? |
|  |  | - [NOT SURE] - Would getting offered HIV and Hep C testing together influence your decision to get tested? |
| *What do you know about Hep C treatment?* | | - What kind of treatments are available? Pills? Injections? - How long does treatment usually last? |
| *Who pays for Hep C treatment?* | |  |
| *Who pays for Hep C treatment in jail?* | |  |
| *If someone is treated for Hep C, they can become reinfected. What do you think about re-treating someone for Hep C who got infection again after getting treated?* | |  |
| *Do you have Hep C?* | | - [YES]: - See “Questions for participants with Hep C” section |
|  |  | - [NO]: - Skip “Questions for participants with Hep C” section |
|  |  | - [NOT SURE:] - Skip “Questions for participants with Hep C” section |
| ***Questions for participants with Hep C:*** | *What has your experience been like with testing and follow-up interaction and care?* |  |
|  | *Thinking back, what things, if any, could have been done differently by health workers to make your testing experience better?* |  |
|  | *Where does treatment for Hep C fall on your list of priorities?* |  |
|  | *If you have received treatment, tell me about the process of getting treated.* | - Who offered treatment? Did you ask for it? Which treatment options were available to you/which option have you received? |
| *Are there reasons why people should be treated for Hepatitis C?* | |  |
| *Are there reasons why people should not be treated for Hepatitis C?* | |  |
| *Is there anything I haven’t asked you about today on this topic that you think is important?* | |  |
| *Do you have any final questions or concerns for me?* | |  |
