# Supplementary material for: “I’m not gonna be able to do anything about it, then what’s the point?”: A broad group of stakeholders identify barriers and facilitators to HCV testing in a Massachusetts jail
Source: PLoS One. 2021 May 26;16(5):e0250901. doi: 10.1371/journal.pone.0250901 (PMC8153419; doi:10.1371/journal.pone.0250901)
Supplement: S2 File — These are the questions asked of participants who were not incarcerated. (DOCX) [file pone.0250901.s005.docx]

**HCV Study: Qualitative Interview Guide:**

**Stakeholder (Non-Inmate, Interview Takes Inside of Jail)**

***Interviewer:***

*Thank you for agreeing to be a part of this interview. I will ask you a series of questions about Hepatitis C Virus (also known as Hep C).*

*There are no “right” or “wrong” answers to your questions. We want to hear anything that you want to talk about. We are going to be typing notes on the computer when you talk and we may ask you to pause or repeat something. We will not put your name on anything that we type or write.*

*If you do not feel comfortable answering a question, it is ok for you to tell me that you do not want to answer. If there’s a question that you’d prefer not to answer, simply say “pass.” You can also choose to stop this interview at any time and for any reason.*

*Your decision to participate or not will not change your health care or the time you are serving in jail.*

*Before we begin, do you have any questions?*

Time Interview Started: ___ ___ : ___ ___ am / pm

Time Interview Ended: ___ ___ : ___ ___ am / pm

Signature of person performing interview: ___________________________________

**HCV Study: Qualitative Interview Guide:**

**Stakeholder (Non-Inmate, Interview Takes Inside of Jail)**

| **Interviewer Asks Verbatim** | **Probes (ask these if the participant needs not sure how to answer or is brief in answer)** |
| --- | --- |
| *Thank you for participating in this study. We are interested in getting to know you and learning about your opinions. First, I would like to start by asking, do you have a favorite song or music artist?* | - Do you remember the first time you heard this song or artist? - Transition: Thank you for sharing that. We look forward to hearing about your opinions. Today we will specifically talk about your thoughts on Hepatitis C. |
| What type of care do you provide for people *in jail?* |  |
| *What have you heard about Hepatitis C?* | - How does someone get Hepatitis C? - When was the first time you heard about Hepatitis C? |
| *How do you feel about Hepatitis C?* | - Does it make you feel differently than HIV? TB? Diabetes? Asthma? |
| *How does someone get tested for Hepatitis C?* | - What kind of tests are there (rapid, serum, viral load, genotype)? - How often should someone get tested for Hep C? |
| *Where does someone get tested for Hepatitis C?* | - Does it need to happen at a primary care office? Emergency room? In jail or prison? |
| *Who pays for Hepatitis C testing?* |  |
| *Where should Hepatitis C testing be offered?* |  |
| *Describe your experience with inmates and Hep C testing?* | - Is Hep C testing offered to everyone? - When is Hep C testing offered? - Who does the test? - What kind of test (viral load, antibody?) - Do you use a blood draw or a finger stick test?  Is Hep C offered with HIV testing? |
| *What kind of training is offered to the people doing the testing?* | - Is there a training for all people about Hep C? - What do you use for educational resources? |
| *What works well about the current process?* | - How can testing be improved? |
| *Are there people who should get tested for Hep C?* |  |
| *Are there people who should not get tested for Hep C?* |  |
| *In your experience, how do inmates feel about Hep C testing?* | - Can you describe any experiences with inmates who either did not want the test? Why do you think they did not want the test? |
| *What barriers do you see to improving Hep C testing?* | - Cost of test, blood draw, staffing?   HIV and Hep C test offered together? |
| *Do you feel comfortable asking people in the jail questions about Hep C?* | - Explain why or why not. Are there certain people you feel more or less comfortable with? |
| *Tell me about HCV treatment* | - What kinds of treatment are available? Pills? Injections? Who should be treated? Does MassHealth pay for treatment? Have you ever seen an inmate get Hep C treatment in jail? |
| *Are there people who should get treated for Hep C?* |  |
| *Are there people who should not get treated for Hep C?* |  |
| *Is jail a good time for Hep C treatment?* | - Why is the jail a good time for Hep C treatment? Why is the jail not a good time for Hep C treatment? |
| *Who are the people who make decisions about Hep C treatment for people in jail?* | - What role does the Sheriff play? How about the Department of Public Health? |
| *Who do you think should pay for Hepatitis C treatment for people in jail?* | - Should the county pay? The Department of Public Health? The criminal justice system? Insurance? |
| *What, if any, changes would you make to this process?* |  |
| *Is there anything I haven’t asked you about today on this topic that you think is important?* |  |
