# Supplementary material for: “I’m not gonna be able to do anything about it, then what’s the point?”: A broad group of stakeholders identify barriers and facilitators to HCV testing in a Massachusetts jail
Source: PLoS One. 2021 May 26;16(5):e0250901. doi: 10.1371/journal.pone.0250901 (PMC8153419; doi:10.1371/journal.pone.0250901)
Supplement: S3 File — These are the questions asked of participants who were not incarcerated. (DOCX) [file pone.0250901.s006.docx]

**HCV Study: Qualitative Interview Guide:**

**Stakeholder (Non-Inmate, Interview Takes Place Outside of Jail)**

Participant ID#: ___ ___ ___

Interview Date: ___ ___ / ___ ___ / ___ ___

M M D D Y Y

Self-Identified Race (eg White, Black, etc):

Self-Identified Ethnicity (Hispanic/Non-Hispanic):

Preferred Language: English Spanish

Age: ___ ___

***Interviewer:***

*Thank you for agreeing to be a part of this interview. I will ask you a series of questions about Hepatitis C Virus (also known as Hep C).*

*There are no “right” or “wrong” answers to your questions. We want to hear anything that you want to talk about. In order to make sure that we have accurate reporting of information from today’s session, I will use this audio recorder to record our conversation. If you do not feel comfortable with me audio recording this interview, we can continue the interview without it being recorded. I will take notes while I ask the questions. I may ask you to pause or repeat something.*

*Is it ok for me to audio record our interview?*

Participant allowed for interview to be audio recorded: YES NO

*If you do not feel comfortable answering a question, it is ok for you to tell me that you do not want to answer. If there’s a question that you’d prefer not to answer, simply say “pass.” You can also choose to stop this interview at any time and for any reason.*

*Before we begin, do you have any questions?*

Time Interview Started: ___ ___ : ___ ___ am / pm

Time Interview Ended: ___ ___ : ___ ___ am / pm

Signature of person performing interview: __________________________________

**HCV Study: Qualitative Interview Guide:**

**Stakeholder (Non-Inmate, Interview Takes Place Outside of Jail)**

| **Interviewer Asks Verbatim** | **Probes (ask these if the participant needs not sure how to answer or is brief in answer)** |
| --- | --- |
| *Thank you for participating in this study. We are interested in getting to know you and learning about your opinions. First, I would like to start by asking, do you have a favorite song or music artist?* | - Do you remember the first time you heard this song or artist? - Transition: Thank you for sharing that. We look forward to hearing about your opinions. Today we will specifically talk about your thoughts on Hepatitis C. |
| *Tell me about the type of services you provide for people that were in jail* |  |
| *How do you feel about Hepatitis C testing and treatment?* | - How does HCV compare to HIV? TB? Diabetes? Asthma? |
| *Are there any differences between testing for Hep C in the community and in a jail or prison setting?* |  |
| *Where should Hepatitis C testing be offered?* |  |
| *What has your experience been like with inmates and Hep C testing?* | - Is Hep C testing offered to everyone? - When is Hep C testing offered? - Who does the test? - What kind of test (viral load, antibody?) - Is Hep C offered with HIV testing? |
| *What kind of training is offered to people testing for Hep C in the community? Do you think it is different for people testing in jail?* | - Is there a training for all people about Hep C? - What do you use for educational resources? |
| *Have you ever been referred a patient who was diagnosed with Hep C in jail? Tell me about this experience* |  |
| *In your experience, how do inmates feel about Hep C-related health care in jail?* |  |
| *What barriers do you see to improving Hep C testing?* | - Cost of test, blood draw, staffing?   HIV and Hep C test offered together? |
| *Is jail a good time for Hep C treatment?* | - Why is the jail a good time for Hep C treatment? Why is the jail not a good time for Hep C treatment? |
| *Who are the people who make decisions about Hep C treatment for people in jail?* | - What role does the Sheriff play? How about the Department of Public Health? |
| *Who do you think should pay for Hepatitis C treatment for people in jail?* | - Should the county pay? The Department of Public Health? The criminal justice system? Insurance? |
| *What barriers (if any) do you see to linking people with Hep C in jail to treatment in the community?* |  |
| *What, if any, changes would you make to this process?* |  |
| *Is there anything I haven’t asked you about today on this topic that you think is important?* |  |
