# Supplementary material for: “I’m not gonna be able to do anything about it, then what’s the point?”: A broad group of stakeholders identify barriers and facilitators to HCV testing in a Massachusetts jail
Source: PLoS One. 2021 May 26;16(5):e0250901. doi: 10.1371/journal.pone.0250901 (PMC8153419; doi:10.1371/journal.pone.0250901)
Supplement: Attachment — Submitted filename: Response to Reviewers- PONE-D-20-35522R1.pdf [file pone.0250901.s007.pdf]

Tufts Medical Center
Proudly wellforce

The principal teaching hospital for Tufts University School of Medicine

800 Washington Street
Boston, Massachusetts 02111
T 617 636-5000
tuftsmedicalcenter.org

March 12, 2021

Dear PLOS ONE Editors,

Thank you for your comments on the paper "I'm not gonna be able to do anything about it, then what's the point?": A Broad Group of Stakeholders Identify Barriers to and Facilitators of HCV Testing in Massachusetts Jails."

Per your request, we have modified the methods section to include the Equator Network guidelines for reporting of qualitative studies (COREQ checklist). We have included a copy of the completed checklist with this submission. We have updated our formatting to align with PLOS ONE's style requirements. We will address other comments from the editors one by one.

Editor Comment 1: Please provide additional information regarding the considerations made for the prisoners included in this study. For instance, please discuss whether participants were able to opt out of the study and whether individuals who did not participate receive the same treatment offered to participants.

Editor Comment 2: Furthermore, please clarify how oral informed consent was documented.

> **Response 1 and 2:** Thank you for this important comment. Yes, people incarcerated in jail were able to not participate in the study. In order to participate, they were asked to give their name to the research team who then consented them in a private setting. The details of procedures are discussed in more detail, page 4, lines 80-92.

Editor Comment 3: Finally when reporting the results of qualitative research, we suggest consulting the COREQ guidelines: http://intqhc.oxfordjournals.org/content/19/6/349. In this case, please consider including more information about: 1) interviewers' training and characteristics ; 2) how participants were selected; 3) if a pilot study was tested; 4) if bias issues were considered. Moreover, please provide the interview guide used as supporting information.

> **Response 3:** Interviewer's training and characteristics have been added (page 5, lines 119-120) a description of how participants were selected (page 4, lines 80-84 and lines 94-103), and a description of the pilot-testing process has been added to the methods section (page 5, lines 113-115). Consideration of bias was discussed in the limitations (page 14, lines 367-374.) We included the interview guides as supplemental information.

Editor Comment 4: In your Data Availability statement, you have not specified where the minimal data set underlying the results described in your manuscript can be found. PLOS defines a study's minimal data set as the underlying data used to reach the conclusions drawn in the manuscript and any additional data required to replicate the reported study findings in their entirety. All PLOS journals require that the minimal data set be made fully available. For more information about our data policy, please see http://journals.plos.org/plosone/s/data-availability. Upon re-submitting your revised manuscript, please upload your study's minimal underlying data set as either Supporting Information files or to a stable, public repository and include the relevant URLs, DOIs, or accession numbers within your revised cover letter. For a list of acceptable repositories, please see http://journals.plos.org/plosone/s/data-availability#loc-recommended-repositories. Any potentially identifying patient information must be fully anonymized. Important: If there are ethical or legal

restrictions to sharing your data publicly, please explain these restrictions in detail. Please see our guidelines for more information on what we consider unacceptable restrictions to publicly sharing data: http://journals.plos.org/plosone/s/data-availability#loc-unacceptable-data-access-restrictions. Note that it is not acceptable for the authors to be the sole named individuals responsible for ensuring data access.

> **Response 10:** We apologize that this was not provided earlier. We needed to consult with the IRB about how to provide this data. Our minimal underlying data set are our codebooks and interview guides. These have been uploaded as supporting information files.

Below are responses to the reviewer comments.

Reviewer 1

Comment 1: Table 1- Could you stratify out clinicians working inside and outside of jail? i.e. specify the number for each of those groups within the provider category?

> **Response 1:** We updated table 1 to delineate providers in each category (Page 5, line 108)

Comment 2: Methods- Please provide more detail regarding the methods, including how you identified study participants in each group. Please refer to the COREQ checklist for reporting and ensure you are reporting all relevant aspects of the study.

> **Response 2:** We have added information on how we identified study groups (page 4, lines 80-84 and lines 94-103). We have also completed the COREQ checklist.

Comment 3: Table 2- Why report these demographic characteristics for people in the jail and why not for other participants?

> **Response 3:** We have amended Table 2 to include age and rage of non-incarcerated participants (Page 6, line 144).

Comment 4:-page 5- In the first paragraph on the section on the stigma theme, you refer to a patient. Should this be participant? Or person in custody? I find this confusing.

> **Response 4:** Thank you for this point. We reviewed the manuscript and tried streamline and clarify the words we use to describe and differentiate the types of stakeholders.

Comment 5 -page 6- Should the title of the theme include both testing and treatment?

> **Response 5**: We agree with this point, and made the change.

Reviewer #2:

Comment 1: Describe in more detail the context for the interviews with persons who are incarcerated. Where were the interviews conducted? Was it a private setting? How did the researcher assistants ensure that potential participants gave fully voluntary and informed consent? Since an IRB defined "vulnerable" population was included in this study, I think the authors should be more transparent about this process for the reader.

Comment 2: Similar to the point above, how were non-incarcerated stakeholders identified for approach?

> **Response 1 + 2:** You bring up a good point, similar to the editor's comments. We have added additional information about the consent process and we clarified the process for identifying and approaching non-incarcerated stakeholders (page 4-5, line 80-107.)

Comment 3: The authors state that two semi-structured interview guides were developed--one for incarcerated persons ("patients") and one for other stakeholders--and discuss how they are similar, but now how they were different?

Response 3: Thank you for this comment. We added additional details about the differences in interview guides to the methods section (page 5, line 111-118).

Comment 4: Minor: similar to how initials were given for the interviewers, can the authors add the initials for the coders

Response 5: We have added the initials for the coders (page 6, line 131).

Comment 5: For the theme "People who are incarcerated frequently did not know that HCV testing is offered in jail," I think the authors should first state whether the Middlesex jail has a policy for testing or not. It seems like there is some ambiguity about testing on the part of jail providers ("One provider who worked in jail reported that HCV testing was not offered to everyone, while others said that it was offered at intake or sick call."). I suggest discussing the HCV policy and practices first, and then the knowledge of the patients. It seems that in addition to not knowing that testing was availably, many patients do not know much about HCV, in general.

Response 5: This is an important point. We added more information to the methods section about the process of HCV testing (page 3-4, lines 75-78).

Comment 6: This may be a personal preference, but I really dislike the headings in the findings. They are too long and too specific. I think that more general headings will better capture the themes that are captured in the data. For example, "stigma against people who use drugs is a barrier to HCV testing" can simply be "Stigma and Deservingness." "Captivity and Transience" can be "Jails as Community Health Care Providers". "Limited Financial Resources Prevents HCV Treatment" becomes Perceived Cost-Effectiveness, etc. The paper is focused specifically on HCV, but I think these themes can likely be generalized to the experience of incarcerated people and healthcare in jails more generally.

Response 6: Thank you for the comment. We have made changes to the headings to keep them broad and shorter.

Comment 7: The final section (Investment in Linkage to Care Programs) seems a little thin, especially compared to the other sections. This theme needs to be fleshed out more.

Response 7: Thank you for this comment. We agree this is an important theme. We have added additional quotes to this discussion (page 10-11, lines 248-267).

Comment 8: The authors did a good job of contextualizing their findings within the current scope of knowledge and literature about HCV testing and treatment. However, as a reader I kept waiting for the list of definitive recommendations. There are suggestions throughout, but I recommend adding a paragraph (either before or after the limitations) that has a clear statement of recommendations for HCV testing and treatment in jails.

Response 8: We appreciate this point, as we hope people can read this and understand the next steps needed to make change. We added a paragraph with recommendations before the limitations paragraph (page 13, lines 349-366).

Comment 9: The authors write "The importance of engaging people who have been incarcerated to help develop HCV testing strategies cannot be overstated. Lived experience can help inform discussions about the best way to communicate preventative healthcare strategies with people who have low health literacy and may lack trust in clinicians working in the jail." This is an important point that seems sort of tacked on. I suggest making this idea its' own paragraph to draw out its importance and maybe discuss ways this has been implemented for other diseases/settings.

Response 9: Thank you for this comment. We have pulled this paragraph out, separated from other points (page 12, 309-317).

Sincerely,

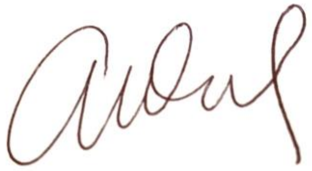

Alysse G. Wurcel MD MS
awurcel@tuftsmedicalcenter.org
617-974-2799
